# Prophylactic Extended-Field Irradiation in Patients With Cervical Cancer: A Literature Review

**DOI:** 10.3389/fonc.2020.579410

**Published:** 2020-10-02

**Authors:** Weiping Wang, Yuncan Zhou, Dunhuang Wang, Ke Hu, Fuquan Zhang

**Affiliations:** Department of Radiation Oncology, Peking Union Medical College Hospital, Chinese Academy of Medical Sciences and Peking Union Medical College, Beijing, China

**Keywords:** cervical cancer, prophylactic extended-field irradiation, para-aortic lymph node, radiotherapy, toxicity

## Abstract

Currently, the standard radiation field for locally advanced cervical cancer patients without evidence of para-aortic lymph node (PALN) metastasis is the pelvis. Due to the low accuracy of imaging in the diagnosis of PALN metastasis and the high incidence of PALN failure after pelvic radiotherapy, prophylactic pelvic and para-aortic irradiation, also called extended-field irradiation (EFI), is performed for patients with cervical cancer. In the era of concurrent chemoradiotherapy, randomized controlled trials are limited, and whether patients with cervical cancer can benefit from prophylactic EFI is still controversial. With conformal or intensity-modulated radiation therapy, patients tolerate prophylactic EFI very well. The severe toxicities of prophylactic EFI are not significantly higher than those of pelvic radiotherapy. We recommend delivering prophylactic EFI to cervical cancer patients with common iliac lymph nodes metastasis. Clinical trials are needed to investigate whether patients with ≥3 positive pelvic lymph nodes and FIGO stage IIIB disease can benefit from prophylactic EFI. According to the distribution of PALNs, it is reasonable to use the renal vein as the upper border of the radiation therapy field for patients treated with prophylactic EFI. The clinical target volume expansion of the node from the vessel should be smaller in the right para-caval region than in the left lateral para-aortic region. The right para-caval region above L2 or L3 may be omitted from the PALN target volume to reduce the dose to the duodenum. More clinical trials on prophylactic EFI in cervical cancer are needed.

## Introduction

Cervical cancer is a major health problem for women in developing countries. In 2015, there were 98,900 new cases and 30,500 deaths in China (1). Para-aortic lymph node (PALN) metastasis plays an important role in the metastasis of cervical cancer. It was reported that 13.5–20.2% of patients with locally advanced cervical cancer (LACC) had positive PALNs (2–5). For patients with FIGO stage IB, IIB and III disease, the incidences of PALN metastasis are 5%, 16% and 25% (4). According to the 2018 FIGO staging system, patients with positive PALNs are allocated to the stage IIIC2 category (6).

At present, the standard treatment for patients with LACC is concurrent chemoradiotherapy. For patients with positive PALNs, extended-field irradiation (EFI) of the pelvis and para-aortic region is recommended. Extended-field intensity-modulated radiation therapy (IMRT) combined with brachytherapy and concurrent chemotherapy is safe and effective for patients with positive PALNs (7, 8). For patients without evidence of positive PALNs, the standard treatment target volume is the pelvis, and the PALN region is not included.

With image-guided brachytherapy, the failure pattern of patients with cervical cancer has changed. And PALN failure is a main failure site. An analysis from the retroEMBRACE study reported that, after image-guided brachytherapy, the pelvic failure, PALN failure, systemic failure and distant (systemic + PALN) failure accounted for 13, 9, 21, and 24% of failure, respectively (9). Another study with EMBRACE study cohort found that, positive LNs at diagnosis are mainly located in the pelvis. However, LNs failure are more often in PALN region. Of patients with nodal failure, 39% were located in PALN region (10). Huang et al. (11) reported that 11% of patients with cervical cancer experienced PALN recurrence after pelvic radiotherapy. In patients with positive pelvic lymph nodes (LNs), the 5-year PALN recurrence rate was 37%. For patients without positive PALNs on imaging, prophylactic EFI has the potential to reduce the incidence of PALN treatment failure after definitive radiotherapy. Currently, whether patients with LACC can benefit from prophylactic EFI is still controversial. In the present article, we review the literature and discuss the necessity, indications, toxicities and target volume contouring of prophylactic EFI in patients with cervical cancer.

## PET/CT in Detecting PALN Metastasis

According to the 2018 FIGO staging system, positive PALNs can be diagnosed with imaging or pathology (6). At present, there is insufficient evidence that surgical staging is beneficial for patients with LACC (12–16). PALN status is determined by imaging for most patients. In meta-analysis, the pooled sensitivities of CT, MRI, and PET/CT were 68, 54, and 71–81%, respectively, in detecting PALN in patients with cervical cancer, and the corresponding specificities were 90, 94, and 97–98%, respectively (17, 18). The positive predictive value and negative predictive value of PET/CT in detecting PALN were 50–100 and 83–100%, respectively (18).

PET/CT has better sensitivity and specificity than CT and MRI in detecting PALN. However, the accuracy of PET/CT in the diagnosis of PALN metastasis is not sufficient, especially for patients with positive LNs. For PET/CT, it was reported that the false negative rate of PALN metastasis was 8.4–13.6% in patients with LACC (19, 20), which is comparatively low. However, in patients with positive pelvic LNs on PET/CT, the false negative rate was 24–27.7% (19, 20), and the negative predictive value of PET/CT in diagnosing PALN metastasis was only 77.6% (5). Ramirez et al. (21) reported that 22% patients with positive pelvic but negative para-aortic LNs on PET/CT had positive histologically confirmed positive PALN.

De Cuypere et al. (5) reported that in patients with LACC, 20.2% had histologic positive PALNs, 10.1% had positive PALNs on PET/CT and 4.8% had positive PALNs both on PET/CT and surgical staging. In patients with positive pelvic LNs on PET/CT, 27.7% had histologic positive PALNs, 19.3% had positive PALNs on PET/CT and 9.6% had positive PALNs both on PET/CT and surgical staging. Imaging modalities, including PET/CT, definitely underestimate the PALN metastasis rate.

The accuracy of PET/CT in the diagnosis of PALN metastasis is not sufficient, and PET/CT underestimate the PALN metastasis rate, especially for patients with positive pelvic LNs.

## Prophylactic EFI Without Concurrent Chemotherapy

Before concurrent chemotherapy became the standard treatment, most patients with LACC received definitive radiotherapy alone. Three randomized controlled trials (RCTs) compared the efficacy and toxicities of pelvic radiotherapy and prophylactic EFI in patients with cervical cancer. In RTOG 79-20,367 patients with FIGO stage IB or IIA (primary tumor ≥4 cm) or with FIGO stage IIB cervical cancer were randomized to the pelvic-only irradiation and prophylactic EFI arms. Conventional radiotherapy was used. In the pelvic-only irradiation and prophylactic EFI arms, the 10-year overall survival (OS) rates were 44 and 55% (*p* = 0.02), respectively, the disease-free survival rates were 40 and 42% (*p* = 0.44), respectively, and the cumulative incidences of first distant failure were 16 and 23% (*p* = 0.053), respectively. Prophylactic EFI tended to increase grade 4–5 toxicities and the death rate due to radiotherapy (22). The other two trials revealed that patients with cervical cancer did not benefit from prophylactic EFI. In the RCT of the EORTC radiotherapy group, compared with pelvic radiotherapy (228 patients), prophylactic EFI (213 patients) did not improve DFS or distant failure and increased the incidence of severe digestive complications (23). An RCT from Japan also revealed that prophylactic EFI did not improve cause-specific survival and distant failure and was associated with more late complications (24). A Cochrane meta-analysis included the three RCTs mentioned above and found that EFI reduced the risk of death (hazard ratio, HR 0.67, 95% confidence interval, CI 0.48–0.94) and PALN recurrence (risk ratio 0.36, 95% CI 0.18–0.70) and that the risk of disease progression was not significantly improved (25).

From 1999–2000, several clinical trials demonstrated that, compared with radiotherapy alone, cisplatin-based concurrent chemoradiotherapy improved the survival of patients with cervical cancer (26–29). Thus, LACC treatment entered the era of concurrent chemoradiotherapy. RTOG 90-01 revealed that pelvic radiotherapy combined with concurrent chemotherapy resulted in OS and DFS rates that were superior to those of prophylactic EFI without concurrent chemotherapy in patients with cervical cancer (30, 31). Clinical trials on prophylactic EFI then stagnated for years.

## Prophylactic EFI Combined With Concurrent Chemotherapy

In the era of concurrent chemoradiotherapy, clinical trials comparing prophylactic EFI and pelvic radiotherapy have been limited. In the study by Asiri et al. (32), 102 patients with FIGO stage IIB-IVA cervical cancer and no evidence of PALN metastasis were randomly assigned to the pelvis concurrent chemoradiotherapy arm (50 patients) and extended-field concurrent chemoradiotherapy arm (52 patients). Seventy-four patients were analyzed. In the pelvic concurrent chemoradiotherapy and extended-field concurrent chemoradiotherapy arms, the PALN control rates were 82.1 and 97.1% (*p* = 0.02), respectively, the distant control rates were 74.7 and 86.9% (*p* = 0.03), respectively, the DFS rates were 69.1 and 80.3% (*p* = 0.03), respectively, and the OS rates were 60.4 and 72.4% (*p* = 0.04), respectively (32). This is the only published RCT (25). Based on the small sample size, imbalance of allocation, and different radiotherapy techniques between the two groups, the outcome of this trial should be interpreted with caution. In the Cochrane meta-analysis, this study was evaluated as having a “high risk of bias” (25).

Some other retrospective studies have compared prophylactic EFI combined with concurrent chemotherapy to pelvic radiotherapy. Lee et al. (33) analyzed 206 LACC patients with negative PALNs. Of these patients, 110 and 96 underwent pelvic radiotherapy and prophylactic EFI, respectively. The upper border of the prophylactic EFI field was at the level of the left renal vessel. In the pelvic radiotherapy and prophylactic EFI patient groups, the 5-year PALN recurrence-free survival rates were 87.6 and 97.9% (*p* = 0.03), respectively, and the OS rates were 74.5 and 87.8% (*p* = 0.04), respectively (33). A retrospective study from our institute included 778 cervical cancer patients without evidence of PALN metastasis. Of these patients, 154 were treated with prophylactic EFI, 624 received pelvic radiotherapy, and 83% received concurrent chemotherapy. After multivariate analysis, prophylactic EFI was an independent prognostic factor of distant failure and PALN failure and not an independent factor of OS or DFS. After propensity score matching, patients treated with prophylactic EFI experienced less distant failure (7.0 vs 21.7%, *p* = 0.016) and PALN failure (0 vs 6.6%, *p* = 0.014) than patients who received pelvic radiotherapy. However, OS and DFS were not significantly different between the two groups (34). In retrospective studies conducted by Yi et al. (35), Oh et al. (36), Park et al. (37), and Yap et al. (38), patients with cervical cancer did not benefit from prophylactic EFI. In the study by Park et al. (37), 203 patients with LACC were included. Prophylactic EFI and pelvic radiotherapy were performed in 88 and 115 patients, respectively. In the pelvic radiotherapy and prophylactic EFI patient groups, the 5-year OS rates were 74.8 and 71.7% (*p* = 0.699), respectively, and the DFS rates were 74.5 and 75.8% (*p* = 0.668), respectively (37). It should be noted that in most retrospective studies, the baseline characteristics were not balanced between the two groups. Patients in the prophylactic EFI group tended to have more advanced disease. For example, in the study by Park et al. (37), more patients in the prophylactic EFI group had positive pelvic LNs (60.7 vs 29.6%, *p* < 0.001), large primary tumors (67.1 vs 42.6%, *p* = 0.002), and more advanced stages (FIGO stage IIIB: 27.3 vs 13.0%, *p* = 0.019) than those in the pelvic radiotherapy group. With this unbalanced baseline, the studies had a high risk of bias.

At present, several clinical trials comparing prophylactic extended-field concurrent chemotherapy and pelvic concurrent chemoradiotherapy have been registered. NCT 00980759/KROG 07-01 is a phase 2 RCT from South Korea. Cervical cancer patients with negative PALNs were randomly assigned to the EFI arm (para-aortic and pelvic irradiation with chemotherapy) and pelvic radiation therapy arm (pelvic irradiation with chemotherapy). The trial started in 2006, and the estimated enrolment number was 312 patients (39). In part 1 of the study, patients were enrolled according to the status of CA9 (hypoxia marker) expression. In part 1, the investigators found that prophylactic EFI reduced PALN recurrence in patients with CA9-positive tumors but that the survival outcome was not improved (40). The final results of the trial have not been released. In 2019, our institute started a multicentre, randomized, phase 3 trial comparing pelvic radiotherapy to prophylactic EFI in selected patients with cervical cancer treated with concurrent chemoradiotherapy in China (NCT03955367) (41). Three other clinical trials, including the NCT 01063387 trial (42), ChiCTR-IPR-14005499 trial (43) and ChiCTR-IIR-17013683 trial (44), were registered. The EMBRACE II study is a prospective study with multiple interventions and multiple endpoints. In this study, nodal clinical target volume (CTV) is defined according to the risk of nodal spread. Patients in the high-risk LNs group will receive pelvis and PALN region irradiation (45).

In the era of concurrent chemoradiotherapy, RCTs are limited, and whether patients with cervical cancer can benefit from prophylactic EFI is still controversial. Thus, more clinical trials addressing prophylactic EFI are needed.

## The Toxicities of EFI

With conventional radiotherapy, the toxicity of prophylactic EFI is unacceptable and much higher than that of pelvic radiotherapy. In the prophylactic EFI and pelvic radiotherapy groups of RTOG 79–20, the incidences of 10-year grade 4–5 toxicities from radiotherapy were 8 and 4% (*p* = 0.06), respectively, and the mortality rates due to radiotherapy complications were 2 and 1% (*p* = 0.24), respectively (22). In the trial of the EORTC radiotherapy group, the 4-year grade 3–4 digestive complications were 8.0 and 3.5% (*p* = 0.005) in the prophylactic EFI and pelvic radiotherapy groups, respectively (23). In the trial from Japan, complications were more frequent in the prophylactic EFI group than in the pelvic radiotherapy group (13/45 vs 2/48, *p* < 0.025) (24). In RTOG 01-16 and RTOG 92-10, EFI was delivered to patients with para-aortic or common iliac LN metastasis with conventional radiotherapy, and the incidence of late grade 3–4 toxicities was 24–40% (7, 46). Based on the high incidence of toxicities, delivery of prophylactic EFI with conventional radiotherapy is not recommended.

In recent years, conformal radiation therapy and IMRT have been used in prophylactic EFI for patients with cervical cancer. In part 1 of NCT 00980759/KROG 07-01, 116 patients were randomized to the prophylactic EFI group or pelvic radiotherapy group. Radiotherapy was delivered with conformal radiation therapy. The acute and late toxicities of patients in the prophylactic EFI group and EFI group were not significantly different. In the prophylactic EFI group, the incidences of grade 3–4 acute upper gastrointestinal, lower gastrointestinal, late gastrointestinal and genitourinary toxicities were 0, 5.3, 3.5, and 1.8%, respectively (40). In the RCT of Asiri et al. (32), radiotherapy was performed with conformal radiation therapy or IMRT. The acute and late toxicities were similar between the pelvic radiotherapy and prophylactic EFI groups. In the study by Lee et al. (47), radiotherapy was delivered with IMRT. The incidences of late ≥grade 3 toxicities in the prophylactic EFI and pelvic radiotherapy groups were not significantly different (3.8 and 2.5%), and no ≥grade 3 genitourinary toxicity was observed in either group. Furthermore, the incidences of acute ≥grade 3 toxicities were also similar between the two groups (47). In our study, the incidences of ≥grade 3 late toxicities were 6.5 and 3.5% in the prophylactic EFI and pelvic radiotherapy groups, respectively (*p* = 0.097) (34). With conformal or IMRT, patients tolerate prophylactic EFI very well. The incidence of severe toxicities from prophylactic EFI is not significantly higher than that from pelvic radiotherapy.

Several prospective randomized trials and one meta-analysis demonstrated that the toxicities of IMRT were lower than that of conformal radiation therapy in patients with LACC treated with definitive radiation therapy (48–51). In the study by Gandhi et al. (51), 44 patients with 2009 FIGO stage IIB-IIIB cervical cancer were randomized to receive whole pelvic conventional radiation therapy or IMRT. Patients in the IMRT arm experienced fewer ≥grade 2 acute gastrointestinal toxicities (31.8 vs 63.6%, *p* = 0.034) and ≥grade 3 gastrointestinal toxicities (4.5 vs 27.3%, *p* = 0.047) compared with patients in the conformal radiotherapy arm (51). A meta-analysis included 6 studies encompassing 1008 patients with cervical cancer who received definitive radiotherapy. Of them, 350 patients treated with IMRT and 658 patients received two-dimensional radiotherapy or three-dimensional conformal radiotherapy. The OS and DFS were not significantly different between the two groups. IMRT significantly reduced ≥grade 3 acute gastrointestinal toxicities (odds ratio, OR, 0.55, 95% CI 0.32–0.95, *p* = 0.03), ≥grade 3 acute genitourinary toxicities (OR 0.31, 95% CI 0.14–0.67, *p* = 0.003), and ≥grade 3 chronic genitourinary toxicities (OR 0.09, 95% CI 0.01–0.67, *p* = 0.02). In the ASTRO clinical practice guideline of 2020, IMRT is conditionally recommended to decrease acute and chronic toxicity in definitive radiotherapy for cervical cancer (52). It should be noted that most patients in these studies are treated with pelvic radiotherapy. However, given the larger target volume and additional organs at risks, IMRT is likely to decrease the risk of toxicities of prophylactic EFI compared with conformal radiation therapy.

## The Indications of Prophylactic EFI

The eligibility criteria differ between studies on prophylactic EFI, and the criteria include cervical cancer (33, 35), LACC (37, 40), FIGO stage IB-IVA (34), FIGO stage IIB-IVA (32), and pelvic LN involvement (36, 40, 47), among others. This is an important reason why these studies have contradictory results. The survival of patients with early-stage cervical cancer is high, with 3-year OS and DFS rates higher than 90% after concurrent chemoradiotherapy (53). For patients with early-stage disease, it is difficult to improve survival with prophylactic EFI. Due to the toxicities of EFI, investigating who could benefit from prophylactic EFI is the main goal now.

Due to the low sensitivity of imaging modalities (17, 18, 54), cervical cancer patients with negative PALNs on imaging may have occult PALN metastasis before treatment. If EFI is not conducted, patients with occult PALN metastasis will experience PALN failure after treatment. Therefore, patients with a high risk of PALN metastasis may benefit from prophylactic EFI. Shim et al. (3) analyzed 245 patients with LACC who underwent para-aortic lymphadenectomy. Of these patient, 34 had pathologically proven positive PALNs. After multivariate analysis, PALN status on PET/CT and tumor size on magnetic resonance imaging were independent predictors of PALN metastasis. A scoring system to estimate PALN status was constructed. PALN metastasis on PET/CT was scored as 5 and 0 for yes and no. Tumor size ≤4 cm, 4.01–5 cm, and >5 cm were scored as 0, 1, and 3 points, respectively. For patients at low (0–1 points), intermediate (3 points) and high risk (=5 points) of PALN metastasis, the predicted probabilities of PALN metastasis were 2.9, 20.8, and 76.2%, respectively (3). Han et al. (55) analyzed 723 patients with FIGO stage IB1-IIA2 cervical cancer who were treated with radical hysterectomy/radical trachelectomy, pelvic LN dissection, and PALN dissection. They found that PALN metastasis was associated with age >46 years, primary tumor size >3.5 cm and FIGO stage IIA (55). In our study based on imaging, PALN metastasis was associated with histology, tumor size, bilateral pelvic LN metastasis and common iliac LN metastasis (56).

As prophylactic EFI reduces PALN recurrence in patients with cervical cancer (32, 34, 47), patients with a high risk of PALN recurrence may benefit from prophylactic EFI. Huang et al. (11) analyzed 758 patients with cervical cancer who were treated with pelvic radiotherapy. After a median follow-up of 50 months, 80 patients had PALN recurrences. In a multivariate analysis, squamous cell carcinoma (SCC) antigen level >40 ng/ml, advanced parametrial involvement and pelvic lymphadenopathy were independent factors associated with PALN recurrence. For patients with SCC antigen >40 ng/ml, advanced parametrial involvement, pelvic lymphadenopathy and no risk factors, the 5-year PALN recurrence rates were 57, 34, 37, and 9%, respectively (11).

The PALN region plays an important role in the distant metastasis of cervical cancer, especially in mediastinal and supraclavicular LN metastasis. Prophylactic EFI may block the pathway of para-aortic lymphatic metastasis and can reduce distant failure in patients with cervical cancer (32, 34, 57). In a previous study, we analyzed 797 cervical cancer patients treated with concurrent chemoradiotherapy and found that non-SCC, common iliac LN metastasis and bilateral pelvic LN metastasis were independent predictors of distant failure. For patients with high-risk (2–3 risk factors) and low-risk (0–1 risk factors) distant failure, the distant failure rates were 39.3% and 19.3% (*p* < 0.001), respectively (58). It was reported that positive pelvic LNs (59–61), advanced FIGO stage (61) and SCC antigen (60) are also associated with distant failure.

In the discussions above, we find that a large primary tumor, advanced FIGO stage, pelvic LN metastasis, bilateral pelvic LN metastasis, common iliac LN metastasis, non-SCC and high SCC antigen levels are risk factors for PALN metastasis before treatment or PALN recurrence and distant failure after treatment. The factors may be used as potential indications of prophylactic EFI. However, this is indirect evidence. These identified risk factors should be validated to determine whether they are indeed predictive of the therapeutic success of prophylactic EFI in patients with cervical cancer. At present, direct evidence on the indications of prophylactic EFI is limited. In the study comparing prophylactic EFI to pelvic radiotherapy by Lee et al. (33), the subgroup analysis revealed that in the pelvic radiotherapy and prophylactic EFI groups, the patients with FIGO III-IVA disease or positive pelvic LNs had 5-year PALN recurrence-free survival rates of 80.1 and 96.4% (*p* = 0.02), respectively, and OS rates of 58.1 vs 83.5%, (*p* = 0.012), respectively. However, these outcomes were not significantly different for patients with FIGO IB-IIB and negative pelvic LNs (33). Another study by Lee et al. (47) analyzed 198 cervical cancer patients with positive pelvic LNs and negative PALN. In the pelvic radiotherapy and prophylactic EFI groups, the patients with positive common iliac LNs or ≥3 positive pelvic LNs had 5-year PALN recurrence-free survival rates of 56.5 and 93.9% (*p* < 0.001), respectively, and cancer-specific survival rates of 56.8 and 100% (*p* < 0.001), respectively. These outcomes were not significantly different in patients with positive pelvic LNs below the common iliac bifurcation and 1–2 pelvic LNs (47). A study from our institute analyzed 133 patients with 2018 FIGO stage IIIB cervical cancer who were treated with concurrent chemoradiotherapy or radiotherapy. In the pelvic radiotherapy and prophylactic EFI patient groups, the 3-year OS rates were 66.3 and 80.3% (*p* = 0.013), respectively, and the DFS rates were 57.2 and 80.4% (*p* = 0.002), respectively (62).

As presented above, common iliac LN metastasis is associated with PALN metastasis before treatment and distant failure after concurrent chemoradiotherapy (56, 58). We have direct evidence that patients with common iliac LN metastasis can benefit from prophylactic EFI (47). In the EMBRACE II study, patients with a positive LNs at the common iliac will be grouped into the high-risk LNs group and will receive EFI (45). In the NCCN guidelines (63) and the review by Jurgenliemk-Schulz et al. (64), EFI is recommended for patients with positive common iliac LNs. Therefore, common iliac LN metastasis is a clear indication of prophylactic EFI. Due to ethical issues, patients with common iliac LN metastasis were excluded from the ChiCTR-IIR-17013683 trial, ChiCTR-IPR-14005499 trial and NCT03955367 trial (41, 43, 44). It has been reported that pelvic LN metastasis is associated with PALN metastasis, PALN recurrence and distant failure (11, 56, 59–61). A retrospective study indicated that prophylactic EFI improved the survival of patients with positive pelvic LNs (33), and thus, patients with positive pelvic LNs may benefit from prophylactic EFI. Because bilateral pelvic LN metastasis is reported to be an independent risk factor of distant failure (58) and because Lee et al. (47) reported that patients with 1–2 positive pelvic LNs below the common iliac bifurcation do not benefit from prophylactic EFI, we recommend that prophylactic EFI can be delivered to patients with ≥3 positive pelvic LNs in clinical trials. In the review by Jurgenliemk-Schulz et al. (64), the authors recommended that prophylactic EFI can be conducted especially for patients with more than two involved LNs. In the EMBRACE II study, >3 positive pelvic LNs serve as an indication of EFI (45). Multiple positive pelvic LNs was also used as an inclusion criterion in the ChiCTR-IPR-14005499 trial (43) and NCT03955367 trial (41). Advanced FIGO stage and parametrial involvement were risk factors for PALN metastasis, PALN failure or distant failure (11, 55, 61). Moreover, prophylactic EFI could improve the survival of patients with 2018 FIGO stage IIIB disease (62). Patients with stage IIIB were included in the NCT00980759, ChiCTR-IIR-17013683, and NCT03955367 trials. Therefore, prophylactic EFI can be delivered to patients with FIGO stage IIIB in clinical trials. A large primary tumor, non-SCC and high SCC antigen were predictors of a high PALN metastasis rate, PALN recurrence and distant failure (3, 55, 56, 58, 60). However, these factors were also associated with worse local control (61, 65, 66). Prophylactic EFI cannot improve the survival of patients with local failure. We do not have direct evidence that patients with these three factors can benefit from prophylactic EFI. Therefore, a large primary tumor, non-SCC and high SCC antigen levels are not recommended as indications of prophylactic EFI.

We recommend delivering prophylactic EFI to cervical cancer patients with common iliac LN metastasis. Clinical trials are needed to investigate whether patients with ≥3 positive pelvic LNs, and FIGO stage IIIB disease can benefit from prophylactic EFI.

## Delineation and Dose Prescription of the PALN Region

Currently, the delineation of the PALN region is inclusive. The delineation of clinical target volume (CTV) is based on the distribution of PALNs. Several studies on the distribution of PALNs are summarized in Table 1.

**TABLE 1:** Studies on the distribution of para-aortic lymph nodes in patients with cervical cancer.

Authors [ref]	Lymph node diagnostic modalities	No. of patients (positive PALNs)	Mean distance from lymph node to adjacent anatomic structures			Distribution of PALNs			
				
			LLPA	AC	PRC	LLPA	AC	PRC	SI direction
Keenan et al. (67)	PET/CT	Design cohort: 21 (39); validation cohort: 10 (29)	8 mm from aorta	8 mm from aorta, 6 mm from IVC	5 mm from IVC	49%	46%	5%	All PALNs were inferior to the left renal vein.
Takiar et al. (68)	PET/CT	30 (72)	8.3 mm from aorta			51%	44%	4%	Inferior third 60%; Middle third 36%; Up third 3%.
			5.6 mm from IVC						
Chao et al. (69)	Lymphangiogram, CT	16	22.1 mm	1.8 mm	9.1 mm				
Kabolizadeh et al. (70)	Size, morphology or PET/CT	46 (133)				59%	35%	8%	

*PALN, para-aortic lymph node; LLPA, left lateral para-aortic; AC, aorto-caval; RPC, right para-caval; IVC, inferior vena cava; SI, superior-inferior; PET/CT, positron emission tomography/computed tomography.*

Generally, the PALN region refers to the area adjacent to the aorta and inferior vena cava from the aortic bifurcation to T12. In the study of Keenan et al. (67), all PALNs were inferior to the left renal vein. Takiar et al. (68) reported that 96% of PALNs were in the inferior and middle third of the PALN region. In the retrospective study by Lee et al. (33), 96 patients received prophylactic sub-renal vein radiotherapy. Only 3 patients (3.1%) experienced PALN failure (34). A smaller CTV results in decreased toxicities. As the incidence of PALN metastasis above the renal vein is low and PALN failure is rare after prophylactic sub-renal vein radiotherapy, it is reasonable to use the renal vein as the upper border of the radiation therapy field for patients treated with prophylactic EFI. In the EMBRACE II study and the review of Jurgenliemk-Schulz et al. (64), the level of renal veins is also used or recommended as the upper border of CTV in patients treated with EFI (45).

PALNs are classified as left lateral para-aortic (LLPA), aorto-caval (AC), and right para-caval (PRC) PALNs based on their location. As shown in Table 1, the mean distances from the LNs to adjacent anatomic structures are 8–22.1 mm in the LLPA region, 1.8–8 mm in the AC region and 5–9.1 mm in the PRC region (67–69). Most PALNs are located in the LLPA and AC regions. Only 4–6% of PALNs are located in the PRC region (67, 68, 70). As LNs in the PRC region are rare and the mean distance from the inferior vena cava to LNs in the PRC region is small, it is reasonable that the node CTV expansion from vessels can be small in the PRC region. Keenan et al. (67) recommended a node CTV expansion of 10 mm circumferentially and 15 mm laterally from the aorta and of 8 mm anteromedially and 6 mm posterolaterally from the inferior vena cava to cover 97% of PALNs. In the study of Chao et al. (69), to cover 100% of lymphangiography-aid PALNs, the node CTV comprised the aorta plus 2 cm and inferior vena cava plus 1 cm and the region 5 mm ventral to the aorta. In addition, PRC LNs are rare above L2 or L3. Takiar et al. (68) reported that all PRC LNs were limited to the inferior third of the para-aortic region (below L3). Keenan et al. (67) found that no PRC lymph node was located above L2 and recommended excluding the RPC region above the L1–L2 interspace. Therefore, the RPC region above L2 or L3 may potentially be omitted from the PALN target volume if the PALN region above L2 or L3 is irradiated. This significantly reduces the dose to the duodenum (71) and may further result in a lower incidence of duodenal toxicities when conducting prophylactic EFI.

In studies which prophylactic EFI was delivered with IMRT or conformal radiation therapy, the dose/fractionation schedules used included 45 Gy in 25 fractions (35, 40, 45), 50.4 Gy in 28 fractions (33, 34), and 45–50.4 Gy in 25–28 fractions (32, 41, 47). NCCN guidelines recommended that coverage of microscopic nodal metastasis requires an EBRT dose of approximately 45 Gy (63). Given that studies comparing different dose/fractionation schedules in prophylactic EFI are lacking, the same dose/fractionation schedule for pelvic nodal coverage (45–50.4 Gy in 25–28 factions) can be used in patients with prophylactic EFI.

## Surgical Staging of PALN Region

Surgical staging and pathologic assessment can provide precise information on PALN status. Given that the accuracy of PET/CT in detecting PALN metastasis remains unsatisfactory (17, 19, 20), surgical staging still has an important role in the era of PET/CT in PALN status assessment.

Compared with imaging staging based CCRT, surgical staging allows accurate adaptation of radiotherapy field. Uterus-11 is a multicenter phase III trial comparing surgical staging followed by CCRT (arm A) and clinical staging followed by CCRT (arm B). A total of 240 patients were eligible, with 121 patients in arm A and 119 patients in arm B. Patients with PALN involvement underwent EFI. Surgical staging resulted in more patients undergoing EFI (23% in arm A vs 12% in arm B, *p* < 0.014) (16). In Uterus-11, surgical staging led to upstaging in 33% of patients (72). De Cuypere et al. (5) reviewed 168 patients with LACC underwent pretreatment PET/CT and PALN dissection. Of them, 35 patients (20.8%) underwent radiotherapy field adaption after PALN dissection. For patients with pelvic LN involvement on PET/CT, 27.7% had radiotherapy field modification. In the study of Gonzalez-Benitez et al. (14), PALN status were assessed with imaging in 31 patients and with surgical staging in 43 patients. In imaging and surgical staging group, EFI was performed in 19.4 and 44.2% of patients (14). In the study of De Cuypere et al. (5) 15 (18.1%) of 83 patients with positive pelvic LNs changed the target volume from the pelvis to the pelvis and PALN region after surgical staging. Ramirez reported that 18.3% of patients had a treatment modification based on surgical findings (21). Mezquita et al. (73) reported that 13.4% of patients had treatment modification to the para-aortic area after surgical staging.

A drawback of surgical staging is the delay of CCRT (13, 74). In Uterus-11, the mean time interval between surgical staging and the beginning of CCRT was 13 days (7–21 days) (72). In the study of Becker et al. (75), the average delay between surgery staging and CCRT was 22 days. Yang et al. (13) reported that the median time duration from diagnosis of cervical cancer to CCRT was 47 days and 28 days in patients treated with surgical staging followed by CCRT and CCRT (*p* < 0.01).

The survival benefit of surgical staging in patients with LACC is still controversial. Lai et al. (12) conducted a randomized trial comparing clinical staging (arm A, *n* = 29) and surgical staging (arm B, *n* = 32) in patients with LACC. Patients receive definitive CCRT or radiotherapy after staging. Patients in arm B had worse PFS (*p* = 0.003) and OS (*p* = 0.024) compared with patients in arm A. The HR of relapse/persistent disease and death (arm B vs arm A) were 1.71 (*p* = 0.006) and 1.50 (*p* = 0.028), respectively. The toxicities between two groups were not significantly different. This trial was stopped after an interim analysis because of the survival reduction of patients in arm B (12). Yang et al. (13) retrospectively analyzed 148 patients with FIGO stage IB2-IIIB cervical cancer treated with CCRT. With propensity score-matching (1:2), 35 patients in the surgical staging group and 70 patients in the imaging group were selected. In surgical staging and imaging groups, the 5-year PFS were 62.6 and 72.4% (*p* = 0.77), the 5-year OS were 70.2 and 70.5% (*p* = 0.96), respectively (13). In the study of Gonzalez-Benitez et al. (14), the mean PFS and OS were not significantly different between patients treated with surgical staging and imaging staging. Gold et al. (15) analyzed 555 patients underwent PALN surgical staging and 130 patients underwent radiographic evaluation from GOG 85, GOG 120 and GOG 165. In patients with stage III-IV disease, the 4-year PFS were 48.9 and 36.3%, and the OS were 54.3 and 40% in surgical staging and radiographic groups, respectively. After multivariate analysis, surgical staging was associated with better PFS (*p* = 0.043) (15). The survival outcome of Uterus-11, a multicenter phase III trial comparing surgical staging followed by CCRT and clinical staging based CCRT, has not been reported (16).

It should be noted that, only patients with positive PALN received EFI in these studies (12, 16, 76), which means that prophylactic EFI was not conducted in patients in imaging group. To our knowledge, prospective studies comparing surgical staging followed by tailored radiotherapy and imaging staging based prophylactic EFI are lacking at present.

Compared with imaging staging based CCRT, surgical staging allows accurate adaptation of radiotherapy field, and leads to the delay of CCRT. It is still controversial whether surgical staging could improve the survival of patients with LACC. And prospective studies comparing surgical staging followed by tailored radiotherapy and imaging staging based prophylactic EFI are lacking at present.

## Conclusion

It is still controversial whether patients with cervical cancer could benefit from prophylactic EFI. With conformal radiation therapy or IMRT, prophylactic EFI is well tolerated. We recommend delivering prophylactic EFI to cervical cancer patients with common iliac LN metastasis. Clinical trials are needed to investigate whether patients with ≥3 positive pelvic LNs, and FIGO stage IIIB disease can benefit from prophylactic EFI. According to the distribution of PALNs, it is reasonable to use the renal vein as the upper border of the radiation therapy field for patients treated with prophylactic EFI. The node CTV expansion from the vessel should be smaller in the right para-caval region than in the LLPA region. More clinical trials are needed on prophylactic EFI in cervical cancer.

## Author Contributions

KH and FZ contributed to the conception of the study. WW and YZ wrote the first draft of the manuscript. DW contributed to the review of literatures. All authors contributed to the manuscript revision, read, and approved the submitted version.

## Conflict of Interest

The authors declare that the research was conducted in the absence of any commercial or financial relationships that could be construed as a potential conflict of interest.
